# A modified quality control protocol for infectious disease serology based on the Westgard rules

**DOI:** 10.1038/s41598-024-67472-1

**Published:** 2024-07-19

**Authors:** Yuanfang Wang, Xiaohan Li, Dongdong Li, Yi Xie

**Affiliations:** Division of Clinical Microbiology, Department of Laboratory Medicine, West China Hospital of Sichuan University, Chengdu, 610041 People’s Republic of China

**Keywords:** Statistical quality control, Asymmetric protocol, Control limits, Infectious disease serology, Westgard rules, Diagnostic markers, Infectious-disease diagnostics

## Abstract

When traditional statistical quality control protocols, represented by the Westgard protocol were applied to infectious disease serology, the rejection limits were questioned because of the high rejection probability. We first define the probability of false rejection (Pfr) and error detection (Ped) for infectious disease serology. QC data in 6 months were collected and the Pfr of each rule in the Westgard protocol and Rilibak protocol was evaluated. Then, as improvements, we chose different rules for negative and positive QC data to constitute an asymmetric protocol, furthermore, while reagent lot changes, the mean value of QC protocol is reset with the first 15 QC results of new lot reagent. QC materials and Standard Reference Materials were tested synchronously in the next 6 months, to verify whether the Pfr and Ped of the asymmetric protocol could meet the requirement. Protocol 1 exhibited the higher level of rejection rate among the two protocols, especially after reagent lot changes; Pfr below the lower control limit (LCL) was 1.39–21.78 times higher than the upper control limit (UCL); false rejections were more likely to occur in negative QC data, with Pfr-total of 27–65%. The asymmetric protocol can significantly reduce the proportion of analytes with Pfr by over 20%. Systematic error due to reagent lot changes and random error due to routine QC data variation were considered potential factors for excessive Pfr. Asymmetric QC protocol that can reduce Pfr by different control limits for negative and positive QC data.

## Introduction

Statistical quality control (SQC) is a widely accepted practice in numerical quality control data, such as clinical chemistry, and is crucial for ensuring the accuracy of patient results. Due to the widespread use of high-throughput automated analyzers and the presentation of test results in numerical form, traditional SQC protocols are now been applied directly to infectious serology testing, which aims to detect antibodies and/or antigens. However, these traditional SQC protocols are problematic and unsatisfactory in infectious disease testing due to significant differences between infectious disease serology and clinical chemistry analytes^1^. In infectious disease serology, immunoassay relies on the coated antigens/antibodies, which may vary among different manufacturers, even within different reagent lots. This lack of consistency reduces the comparability and traceability of quality control (QC) results across manufacturers. Additionally, changes in serotypes, genotypes, or mutations in pathogens contribute to antigen heterogeneity, making the antigen–antibody reaction more complicated^1–3^. Furthermore, QC results in infectious disease serology are often presented in a qualitative or semiquantitative manner, making them untraceable and inaccurately quantifiable^3,4^. These results also fail to account for systematic differences that may arise from changes in reagent lots. Therefore, the direct application of traditional SQC protocols to infectious serology lead to high rate of false rejection^5^, it has become a prevalent phenomenon that triggers costly and unnecessary investigations^6^.

Typically, when a new quality control material adopts, it is necessary for the obtained QC results to fall within the range specified by the manufacturer. However, during subsequent quality control result monitoring, the SQC protocol specified in the guidelines will be used to make a more precise assessment of the QC results. At this point, if the QC results do not comply with a specific rule in the QC protocol, they need to be evaluated, regardless of whether they fall within the broad range specified by the manufacturer. As the most used SQC protocols, the Westgard protocol is the most classical multipole rule protocol^7–10^, while the RiliBÄK protocol is a single rule protocol which utilizes a single rejection limit or control interval to determine whether to reject^11,12^, both of them establish independent mean values and rejection limits for each lot of QC material. Compared to the Westgard protocol, RiliBÄK protocol is simpler for determining rejection and typically yields a lower rate of rejection in infectious serology^5^, but it also can’t recognize true or false rejection. Indeed, only a limited number of prior studies have focused on quality control protocols for serology^5,13–17^, the majority of them demonstrated that the available SQC protocols were not a suitable option for infectious serology. To address the issue of overly tight control limits, some studies have suggested the use of longer periods of QC data from different lots to create pooled CV and establish broad rejection limits, similar to The QConnect^™^ protocol, which incorporates the reagent lot as part of the variation in the detection process, validation procedures were executed upon the transition of reagent lots^18^. However, building pooled CV of different reagent lots is at risk that the control limits are too wide to detect a true errors of clinical sample result. Simultaneously, power function graphs, critical-error graphs, and the sigma-metric QC planning tool system^19^, which are usually widely used for quantitative biochemical QC data, cannot be directly applied to numeric QC data for infectious diseases. Currently, how to determine the probability of false rejection (Pfr) for infectious diseases SQC protocols and how to select the appropriate QC rules, are questions that need to be addressed. Based on a study from Dimech et al.^5,18^, our study complements the definition of false rejection of infectious serology SQC protocols, evaluates Pfr and explores why false rejection occurs. Most importantly, we propose a modified protocol that can further reduce the Pfr of infectious serology SQC protocols.

## Materials and methods

### Definitions

In this study, QC data sets with the same QC material lot were used to evaluate the performance of the QC protocol. When a rejection occurs, it is documented and classified as either true or false. For the retrospective part of this study, a rejection is considered a true rejection if the negative QC material yielded a reactive result or the positive QC material yielded a non-reactive result. Rejections occurring in other cases were considered false rejections and are used to calculate Pfr. In the prospective part of this study, as well, standard reference materials (SMRs) were also simultaneously tested as a reference to determine whether a rejection is false or not. When SMRs in control, QC data sets that exceed the rejection limits were considered false rejections. This approach helps to assess the accuracy and reliability of the QC protocol in detecting true errors and minimizing false rejections, and it is a preferred method for comparing reagent lot-to-lot variation causes of its stability^1^.

In the Westgard rules, the total Pfr is defined as the proportion of triggering any one or more of the three subrules (1-3 s, 2-2 s, and R4s). Pfr_high_ represents the proportion of QC data that exceed the upper control limit, while Pfr_low_ represents the proportion of QC data that fall below the lower control limit.

The formula is:

$$\text{Pfr}_\text{total}({\%})=\frac{{N}_{1-3s}+{N}_{2-2s}+{N}_{R4s}}{{\text{N}}_{\text{total QC }} }\times 100\%$$

In our study, ‘nSD’ is used to quantify the distance between the means of the first 20 results on the Levey–Jennings chart before the reagent lot change $\left({\overline{x} }_{pre}\right)$ after the reagent lot change $\left({\overline{x} }_{after}\right)$. ‘n’ is used to quantify the fluctuation of the QC data when the reagent lot changed, a larger value of nSD indicates a greater degree of variation in the QC data. The formula is:

$$n\text{SD}=\frac{\left({\overline{x} }_{pre}-{\overline{x} }_{after}\right)\times {CV}_{pre}}{{SD}_{pre}}$$

### Traditional protocols and the asymmetric protocol

Here, the traditional protocols are divided into two protocols for further analysis. Protocol 1 is based on Westgard rules, and twenty QC results are used to establish mean and SDs; Protocol 2 is based on the RiliBÄK protocol, which only using mean value plus or minus deviation limits (${\Delta }_{max}$) as upper and lower control limits, to judge whether QC result should be rejected. The equation ${\Delta }_{max}=\sqrt{{K}^{2}\times {S}_{ep}^{2}+{\updelta }_{ep}^{2}}$ was used to calculated ${\Delta }_{max}$, with the first fifteen QC results, K is the coverage factor for calculating the internal laboratory deviation limits, usually set into 3 according to the guideline, S_ep_ is the empirical SD of the QC material measurements used in the calculations, given that infectious disease serology QC samples do not have a “true value”, for the purposes of this study, $\updelta$ will not be taken into consideration, the equation ${\Delta }_{max}=\sqrt{{K}^{2}\times {S}_{ep}^{2}}$ was used^5,11,20^.

The asymmetric QC protocol utilized in our study is set as follows: the mean value ($\overline{x }$) is determined by calculating the average of the first fifteen QC data. For negative QC material results, the upper control limit (UCL)$\text{is defined as }\overline{x }+{\Delta }_{max}$, while no lower control limit (LCL) is established; that means negative QC results are only rejected if they exceed the UCL. The formula for Δmax was also $\sqrt{{K}^{2}\times {S}_{ep}^{2}}$, and Sep is the standard deviation of fifteen QC data^5,11^. The positive QC data follow the Westgard rules of 1-3 s and 2-2s^7^. Whenever a reagent lot changes, the mean and standard deviation are recalculated using the first fifteen QC data points from the new lot. Supplementary Table 1 provides more comprehensive details on these three protocols.

### Assays and instruments

Hepatitis B surface antigen (HBsAg), hepatitis C antibody (A-HCV), syphilis antibody (A-TP), and HIV Duo consisting of HIV p24 antigen (HIV Ag) and HIV antibody(A-HIV) were included in the study. All analytes were measured by all of four Cobas e801 modular (Roche Diagnostics, Germany), using the electrochemiluminescence assay (ECLIA). Commercial QC materials for these five analytes were routinely provided by Roche Diagnostics. SMR and negative serum QC materials were provided by Beijing Conchstein Biotechnology Co. Ltd. (more detailed information on the materials is provided in Supplementary Table 2. The SMR is presented in a powdered state, subsequently dissolved and configured in accordance with the specifications provided by the manufacturer, and subjected to concurrent testing alongside quality control materials.

### Study design

The Pfrs were initially calculated retrospectively. All the QC data of HBsAg(*N* = *2505*), A-HCV (*N* = *2196*), A-TP (*N* = *1832*), HIV P24 antigen (HIV Ag, *N* = *2646*), and HIV antibody (A-HIV, *N* = *2646*) from January to June 2021 were extracted from our laboratory. Subsequently, from Aug 2021 to Jan 2022, SMRs of the five analytes were performed simultaneously as a criterion to determine the authenticity or false rejection of the QC data in a prospective manner. Supplementary Table 3 provides information on the lots and data amount of QC and SMR that are included in each analysis.

The probability for error detection (Ped) for all QC protocols was evaluated by simulating data in our study. In this simulation, 200 data within the range of 0.8–1.0 cut off index (COI) or 1.0–1.2 COI were generated. These data were classified as critical negative and critical positive results, respectively (Supplementary Table 4, 5 and6). The critical positive data were used to verify the Ped of negative QC rules, while the critical negative results were used to verify the Ped of positive QC rules. When the critical result is correctly identified as rejected by a QC rule, it means that the rule has successfully detected an error. On the other hand, if a critical result is not rejected, it means that the rule has failed to detect the error. The ratio between the correctly detected and incorrectly detected critical results is used to calculate the Ped. Based on the assumption that the QC rules can correctly judge the rejection of critical results, it can be inferred that they can also judge the rejection of non-critical results.

### Statistical methods

We calculate Pfr of each dataset with Microsoft Excel 16.0 and use a t-test to compare Pfr of different instruments and analytes. All data analyses and simulations are performed using IBM SPSS Statistics 26, and *P* < 0.05 is considered to indicate statistical significance.

### Ethics

This work was already approved by the Ethics Committee of West China Hospital of Sichuan University (No.920), this study deal only with quality control data and not with patient sample results.

## Results

### Pfr estimation of traditional protocols

In the retrospective study, a total of 96 datasets were collected from four instruments, focusing on five analytes. Table 1 presents the false rejection rates (Pfr) of two traditional protocols. The Pfr_total_ for the five assays in Protocol 1 ranged from 26 to 51%, with a median of 43%, which was higher in the two protocols. In Protocol 2, the Pfr ranged from 7 to 42%. Further analysis of the three subrules of Protocol 1 revealed that the 2-2 s rule was frequently triggered, while R-4 s rule was rarely triggered (Table 1). Across all protocols and subrules, it was observed that false rejections above the UCL (Pfr_high_) were significantly less likely to occur compared to those below the LCL(Pfr_low_). The variance range between Pfr_high_ and Pfr_low_ was1.39–21.78-fold with a median of 2.24-fold. This finding serves as a reminder that setting the lower control limit too high may contribute to false rejections.

**Table 1:** Pfrs for each protocol of each analyte.

	Number of results	Failure above or below the acceptance range	Pfr of Protocol 1% (number)	Pfr of subrule of Protocol 1%(number)				Pfr of Protocol 2% (number)	*P* value of three rules
				1-3 s	2-2 s	R4-s	*P* value of subrules		
HBsAg	2505	high	19 (468)	12 (240)^a^	15 (313)^b^	3 (59)^c^	< 0.05	10 (253)	< 0.05
		low	7 (186)	9 (184)^a^	7 (151)^a^	3 (71)^b^	< 0.05	6 (159)	0.015
		total	26 (654)	21 (424)^a^	23 (464)^a^	6 (130)^b^	< 0.05	16 (412)	< 0.05
A-HCV	2196	high	10 (214)	0 (4)^a^	1 (16)^b^	0 (6)^a,b^	0.263	0 (7)	< 0.05
		low	44 (944)	34 (823)^a^	45 (980)^b^	6 (140)^c^	< 0.05	7 (153)	< 0.05
		total	54 (1178)	38 (827)^a^	45 (996)^b^	7 (146)^c^	< 0.05	7 (160)	< 0.05
A-TP	1832	high	16 (284)	10 (188)^a^	11 (200)^a^	2 (28)^a^	0.262	14 (261)	0.091
		low	35 (645)	22 (519)^a^	34 (624)^b^	1 (25)^c^	< 0.05	28 (506)	< 0.05
		total	51 (929)	39 (707)^a^	45 (824)^b^	3 (53)^c^	< 0.05	42 (766)	< 0.05
A-HIV	2646	high	20 (538)	17 (448)^a^	18 (469)^b^	4 (105)^b^	< 0.05	20 (531)	0.085
		low	15 (388)	12 (322)^a^	17 (437)^b^	1 (34)^c^	< 0.05	8 (213)	< 0.05
		total	35 (926)	29 (770)^a^	37 (906)^b^	5 (139)^c^	< 0.05	28 (744)	< 0.05
HIV Ag	2646	high	29 (771)	17 (446)^a^	16 (431)^a^	3 (92)^b^	< 0.05	21 (551)	< 0.05
		low	14 (361)	8 (214)^a^	11 (299)^b^	3 (67)^c^	< 0.05	15 (386)	0.065
		total	43 (1132)	25 (660)^a^	28 (730)^a^	6 (159)^b^	< 0.05	36 (937)	< 0.05

Analytes with different subsequent letters (a,b, and c)of Pfr were considered statistically significant differences at the 0.05 level.

Since Protocol 1 exhibited the highest Pfr, the Pfr of negative and positive QC datasets were further assessed separately within Protocol 1 (Table 2). It was found that negative QC data had a higher likelihood of being rejected, with a Pfr_total_ reaching up to 65%, On the other hand, the Pfr-total of positive QC data did not exceed 43%. Consistent with the previous results, the R4-s rule was the least frequently triggered in both negative and positive QC data sets. For negative QC material, false rejections were more likely to occur in data below the LCL. Conversely, most false rejections in positive QC data observed in results that exceeded the UCL.

**Table 2:** Pfr of negative/positive QC material for each analysis.

	Failure above or below the acceptance range	Protocol 1	Pfr of negative control %(number)			*P*-value of three subrules	Protocol 1	Pfr of positive control %(number)			*P*-value of three subrules
			1-3 s	2-2 s	R4-s			1-3 s	2-2 s	R4-s	
HBsAg	high	16 (164)	12 (125)^a^	6 (60)^b^	4 (36)^c^	< 0.05	30 (304)	20 (207)^a^	25 (253)^b^	2 (23)^c^	< 0.05
	low	18 (186)	5 (51)^a^	15 (151)^b^	3 (29)^c^	< 0.05	7 (75)	4 (41)^a^	3 (35)^a^	4 (42)^a^	0.685
	total	27 (275)	17 (176)^a^	17 (176)^a^	6 (65)^b^	< 0.05	37 (379)	24 (248)^a^	28 (288)^a^	6 (65)^b^	< 0.05
A-HCV	high	1 (11)	0 (3)^a^	0 (1)^a^	0 (5)^a^	0.263	1 (16)	0 (1)^a^	1 (15)^b^	0 (1)^a^	< 0.05
	low	65 (713)	53 (582)^a^	62 (683)^b^	1 (6)^c^	< 0.05	41 (452)	22 (244)^a^	27 (298)^b^	12 (134)^c^	< 0.05
	total	64 (710)	53 (582)^a^	62 (683)^b^	1 (11)^c^	< 0.05	43 (468)	22 (245)^a^	29 (313)^b^	12 (135)^c^	< 0.05
A-TP	high	1 (5)	0 (3)^a^	0 (1)^a^	1 (5)^a^	0.262	31 (279)	20 (185)^a^	28 (256)^b^	2 (19)^c^	< 0.05
	low	62 (568)	52 (482)^a^	57 (525)^a^	2 (15)^b^	< 0.05	8 (77)	4 (37)^a^	5 (42)^a^	1 (10)^b^	< 0.05
	total	62 (573)	53 (485)^a^	57 (526)^a^	3 (24)^b^	< 0.05	39 (356)	24 (222)^a^	33 (298)^b^	3 (29)^c^	< 0.05
HIV Ag	high	25 (216)	14 (127)^a^	18 (154)^a^	1 (10)^b^	< 0.05	27 (240)	19 (169)^a^	22 (196)^a^	2 (21)^b^	< 0.05
	low	17 (153)	11 (95)^a^	17 (149)^b^	1 (9)^c^	< 0.05	10 (86)	6 (51)^a^	8 (69)^a^	2 (15)^b^	< 0.05
	total	42 (369)	25 (222)^a^	36 (313)^b^	2 (19)^c^	< 0.05	37 (326)	25 (220)^a^	30 (265)^a^	4 (36)^b^	< 0.05
A-HIV	high	18 (161)	15 (129)^a^	11 (95)^b^	6 (54)^c^	< 0.05	28 (251)	24 (214)^a^	26 (234)^a^	2 (19)^b^	< 0.05
	low	24 (211)	14 (121)^a^	20 (176)^b^	2 (18)^c^	< 0.05	7 (62)	4 (39)^a^	4 (38)^a^	4 (31)^a^	0.577
	total	42 (372)	28 (250)^a^	31 (271)^a^	8 (72)^b^	< 0.05	35 (313)	29 (253)^a^	31 (272)^a^	6 (50)^b^	< 0.05

Analytes with different subsequent letters (a,b, and c) of Pfr were considered statistically significant differences at the 0.05.

### Possible factors for high Pfrs

**Figure 1:**
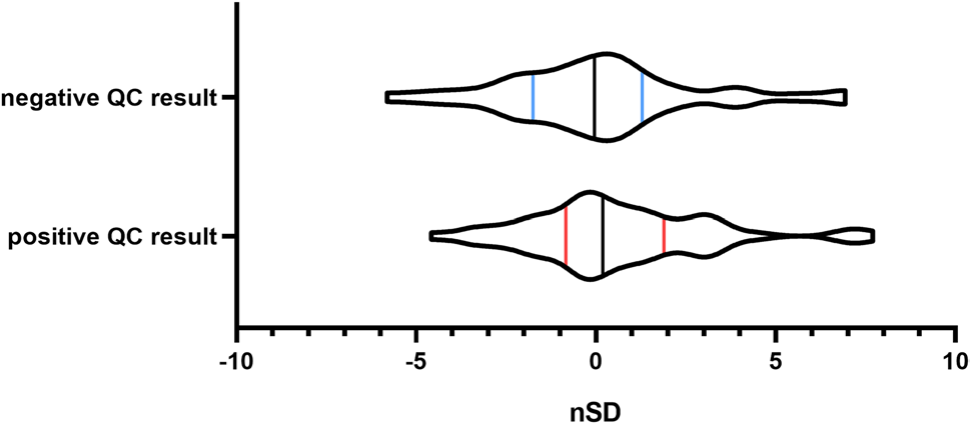
nSD after reagent lot change The nSD distribution of positive and negative QC results show as a violin diagram. The number of positive QC results was frequent at + 3SD, which indicates that positive QCs are more likely to be rejected.

The focus of our investigation was directed towards the impact of reagent lot changes, which have the potential to induce system errors. Within the retrospective dataset, a total of 88 reagent lot changes were identified (Supplementary Table 6), Notably, 65% (57/88) of nSD after reagent lot changes exhibited values exceeding one, thereby necessitating the redefinition of mean values. Specifically, 36% (32/88) of these changes were occurred in negative QC materials and 28% (25/88) were observed in positive materials. Furthermore, 25% (22/88) of the results displayed more than 3SD from the mean subsequent to reagent lot changes, with 14% (12/88) occurring in negative materials and 11% (10/88) in positive materials. Notably, the nSD value reached a maximum to 7.71 due to the reagent lot changes, ultimately lead to false rejection. The distribution of nSD values for positive QC data spanned from -2SD to 4SD, while negative QC data exhibited a narrower distribution within the range of ± 2SD. This observation suggests that positive QC results are more susceptible to false rejections in the presence of reagent lot changes (Fig. 1).

**Figure 2:**
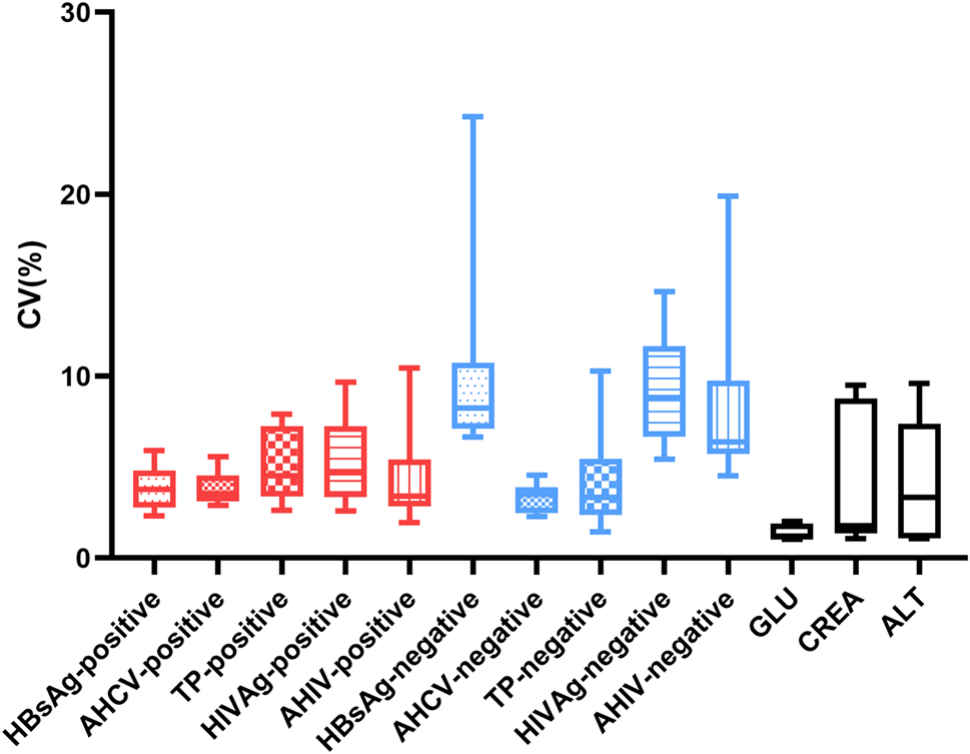
CV distribution of the QC dataset. The CVs of QC results for a total of ten QC materials for five different analyses are presented using box plots, each box plot representing the distribution of CV results for all data sets for one QC material for one analysis. Dots represent an outlier of the CV data, which is more often found in negative material.

High variability in routine QC data, which may lead to random error, was also considered as a potential factor contributing to the elevated Pfr. It was found that 65% (62/96) of datasets had CVs exceeding 5%, indicating poor imprecision and a higher probability of rejection. The CV distribution of negative and positive QC datasets was shown in Fig. 2. It was observed that the median CV of the three negative QC datasets exceeded 5%, including HBsAg, HIV Ag, and AHIV, while all median CVs of the five positive materials were less than 5%. Significant differences showed between instruments and reagent lots, with variations reaching up to 7.05-fold (Supplementary Fig. 1, 2, 3, 4, 5, 6, 7, 8, 9 and 10), and finally resulting in differences in Pfr between instruments (Supplementary Table 7).

### The asymmetric QC protocol

Based on the aforementioned studies, a selection was made to utilize different QC rules for negative and positive QC datasets based on the Westgard protocol, resulting in the development of an asymmetric QC protocol. In order to confirm the optimal number of QC data required for establishing new mean values, three datasets containing over 150 QC data points were utilized (positive material for HBsAg, AHCV, and A-TP). Control limits were established with 15, 20, 40, 60, 80, and 100 QC results, respectively, with the aim of assessing whether different inclusion numbers would result in different CVs and necessitate adjustments to the control limits. As a result, the increase in CV was not statistically significant after using a higher number of QC data (*P* = 0.558) (Supplementary Table 8^8^. This suggested that even when 100 QC data points were employed to establish the mean value, the acceptance range was not wider than that obtained when 15 QC data were used, and thereby obviating the need for subsequent limit adjustments over time.

### Pfr and Ped estimation of the asymmetric QC protocol

**Figure 3:**
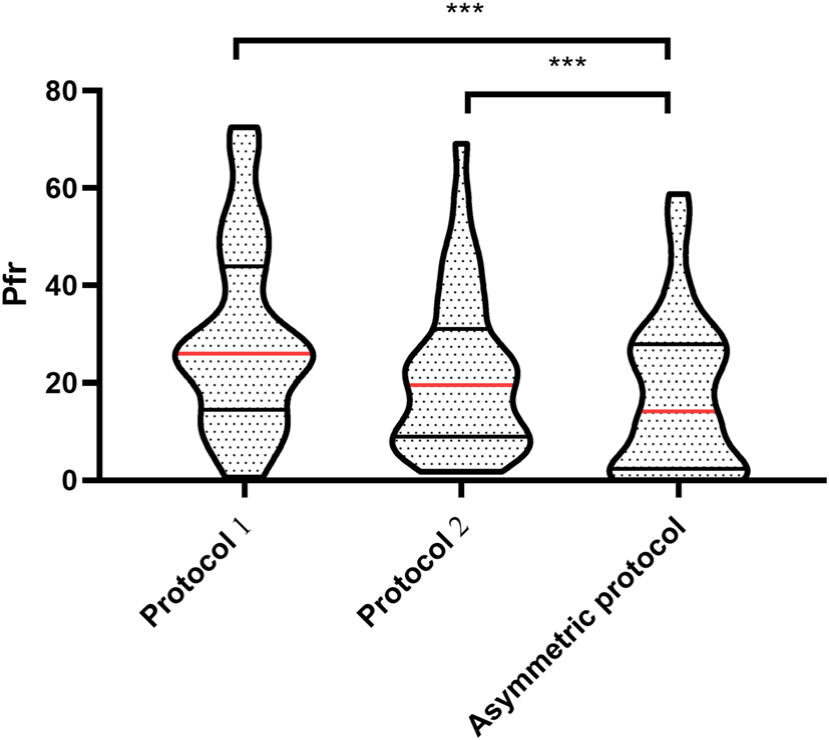
Pfr distribution for different QC protocols The different color blocks represent the Pfr of the different QC protocols respectively. A higher mean line implies a high Pfr and a wider box plot suggests a higher variation in Pfr.

In the prospective analyses part, a total of 46 QC datasets were obtained and evaluated for Pfrs with two traditional protocols and the asymmetric QC protocol in different analyses and instruments were (Supplementary Table 9). The median Pfr of Protocol 1, Protocol 2, and the asymmetric protocol were 26%, 20%, and 15%, respectively. It is generally accepted that a Pfr of less than 5% is considered acceptable in clinical laboratories, In the new model,13 out of the 40 Pfrs in the new model (33%) met this requirement, whereas only 3 and 4 of the 40 Pfr analyses could meet this requirement with Protocol 1(8%) and Protocol 2(10%) respectively. The decrease in Pfr for negative QC datasets was found to be significant, and HBsAg was the most suitable for this protocol. Proportions of high Pfr (Pfr > 20%) with the asymmetric QC protocol was only 43% (17/40), which was lower than the proportions observed with Protocol 1(70%,28/40) and Protocol 2(50%,20/40). Figure 3 visually demonstrated the significant reduction in Pfr achieved by the asymmetric protocol.

Simulated data was utilized to analyze the probability of error detection (Ped) for the three protocols (Supplementary Table 10), the results revealed that all three protocols were able to correctly identify 100% of the critical results as rejections. Furthermore, there was no discernible difference in the ability of the different types of QC protocols to detect qualitative errors in the QC results.

## Discussion

Serological tests are used to differentiate individuals with specific antibodies or antigens from those without, by providing an appropriate numeric COI or signal-to-cutoff ratio(S/CO) result. In general, the S/CO between non-reactive and reactive results may not be linear, which is completely different from the standardized and traceable detection of biochemistry^18^. Therefore, commutability between the QC and patient sample results is typically poor, and rejection of QC results rarely signifies incorrect patient sample results. At present, a high rejection rate is not clinically significant and may interfere with clinical practice. As acknowledged by the WHO Expert Committee on Biological Standardization, the development of international standards for quantitative immunoglobulin measurement standard efforts has been unsuccessful^21^. Therefore, it is necessary to establish a QC protocol that is better suited to the characteristics of serological testing.

In our study, the adequacy of the traditional and asymmetric QC protocols was evaluated using the Pfr as the primary metric. Pfrs in the two traditional protocols often exceeded 20%, particularly in the Westgard protocol, which was deemed too high for clinical practice. The triggering of the 2-2 s subrule multiple times during lot conversion often resulted in an incorrect mean value and a significant increase in Pfr. However, it did not lead to errors in sample results. In contrast, the triggering of the R-4 s subrule was rare, indicating that a change in the distribution range of the QC data and representing a change in the entire system rarely happen^22^, and this subrule usually occurs only once when the reagent lot changes. The RiliBÄK protocol exhibited the lowest Pfr, with a single rejection limit of mean ± Δmax, suggesting that reagent lot changes usually did not result in false rejections.

Furthermore, more false rejections occurred in the negative QC data. The negative QC materials were representative of the noise in the testing process, highlighting that the LCL of negative QC materials is of little value. According to the patient risk based quality control concept^7^, negative QC data below the LCL will not result in misjudgment of patient results. Hence, the rules from the RILIBAK protocol have been chosen as a reference for negative QC datasets in the asymmetric protocol, and the Westgard protocols have been enriched in serological testing.

Similar to Wayne J Dimech et al.^1^, QC results are shifted when changing reagent lot, the nSD is used to quantify this shift, and it was up to nearly 8, fluctuation in QC results is caused by the changing of reagent lot, which raises unexpected false rejections, it’s beyond the usual perception, is highly unlikely that changes in reagent lots significantly contribute to alterations in the clinical sensitivity or specificity of infectious serology tests^5^.Similar to quantitative testing^23^, there were significant differences in CV between different instruments and analytes, which can be attributed to inherent properties of the instrument and characteristics of the serologic assay itself. This results in significant differences in the Pfrs between instruments or analyses. In fact, there were numerous other sources of variance in QC data, including instrument calibration and maintenance, operating procedures, storage and shipping conditions of reagents and consumables, and environmental conditions^15,24^. These factors were not addressed in our study.

Recent CLSI guidance suggests that rejection limits should be redefined once after three months of data cumulating with more than 100 data points^7^, but does not specify the exact number of QC data required. However, as we know, the validity period of serological QC materials is usually not long enough, making the data accumulation more difficult. Some opinions suggest that with an increase in cumulative data, the increasing CV of QC data may make the control intervals becoming too wide to make a correct rejection^25^. We demonstrated that the differences in cumulative CV from the first 15 points to the first 100 QC points were not statistically significant, and simply increasing the number of QC data may not reduce false rejections. Therefore, we concluded that determining the mean value and SD with the first 15 QC points may be a good choice for clinical practice.

Hence, optimization and supplementation of the Westgard protocol are necessary for its application to serological testing. The Asymmetric QC protocol may be considered a more appropriate option. By setting only UCL ($\overline{x }+{\Delta }_{max}$)for negative QC datasets and still using some of rules from Westgard protocol for positive QC datasets, could successfully reduce Pfr. It is important to note that it is necessary to reaccumulate the mean values and use the CV of the old QC reagent lot to determine the SD, which could solve the problem of poor comparability between lots and reduce Pfr during lot change. The number of QC data points used for cumulative mean values has been reduced to 15 points, which allows for faster determination of appropriate mean values, reducing labor and reagent consumption. The evaluation of Pfr and Ped for asymmetric QC protocols also demonstrated that the reduced QC rules and loosened control limits of the protocol did not affect the ability to detect a true rejection.

In conclusion, the traditional SQC protocols for infectious disease serology with numeric QC data exhibit unacceptably high Pfr. Our study proposed an asymmetric QC protocol, as an improvement to the Westgard protocol, which is a nice attempt that can successfully reduce the possibilities of false rejections. We demonstrated that the asymmetric QC protocol leads to lower Pfrs in more analytes and instruments, and it is a well-tried application of the Westgard protocol to serological testing.

## Limitation

Our study included only five infectious disease tests from four instruments in our laboratory, and the amount of data was not rich enough. In addition, limited by the laboratory situation, we ended up using simulated data, rather than real test results, to evaluate Ped, and this part of the results has some predictive significance, but further studies are needed for confirmation. Bias may exist due to potential errors in evaluating Pfrs and Peds.

### Supplementary Information

Supplementary Figures.

Supplementary Tables.

## Data Availability

All data used for this study are contained in this article.

## Abbreviations

SQC: Statistical quality control
QC: Quality control
COI: Cut-off index
Pfr: Probability of false rejection
Ped: Probability of error detection
SD: Standard deviation
CV: Coefficient of variation
S/CO: Signal to cut-off
