# Supplementary material for: A modified quality control protocol for infectious disease serology based on the Westgard rules
Source: Sci Rep. 2024 Jul 19;14:16683. doi: 10.1038/s41598-024-67472-1 (PMC11271505; doi:10.1038/s41598-024-67472-1)
Supplement: Supplementary file 1 — Supplementary Figures. [file 41598_2024_67472_MOESM1_ESM.pdf]

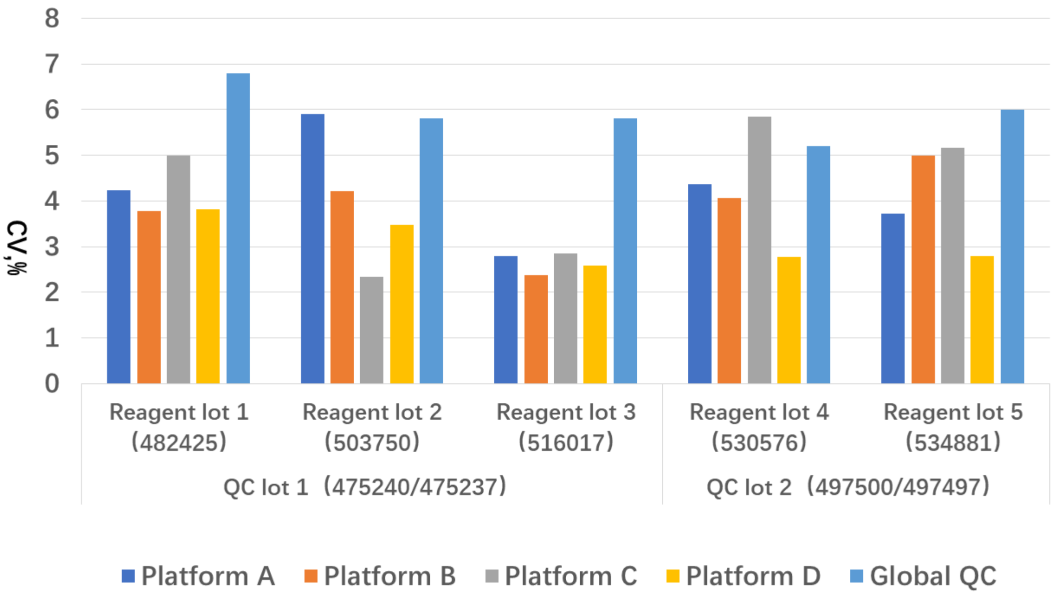

Supplementary Figure 1 CV for HBsAg positive QC material and reagent lot partition for each instrument over a 6-month interval.

The CV variation was 1.48-2.51-fold between five reagent lots of the same instrument for HBsAg positive QC, 1.33-2.10-fold between the same reagent lot for four instruments, and 2.51-fold between different reagent lots and different instruments. The CVs of reagent lot 2 for instrument A and reagent lot 4 for instrument B were higher than the CVs of the global QC data for the same lot.

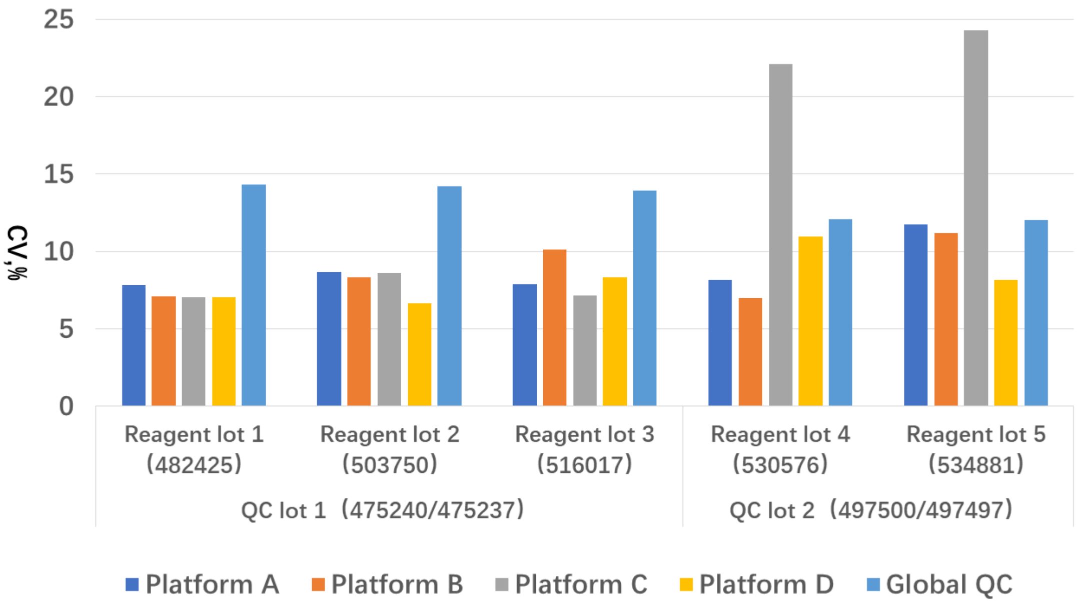

Supplementary Figure 2 CV for HBsAg negative QC material and reagent lot partition for each instrument over a 6-month interval

The CV variation was 1.11-3.26-fold between five reagent lots for the same instrument for the HBsAg negative QC, 1.51-3.49-fold between the same reagent lot for the four instruments, and 3.70-fold between different reagent lots and different instruments. The

CVs of reagent lot 4 and reagent lot 5 for instrument C were higher than the CVs of the same lot of global QC data.

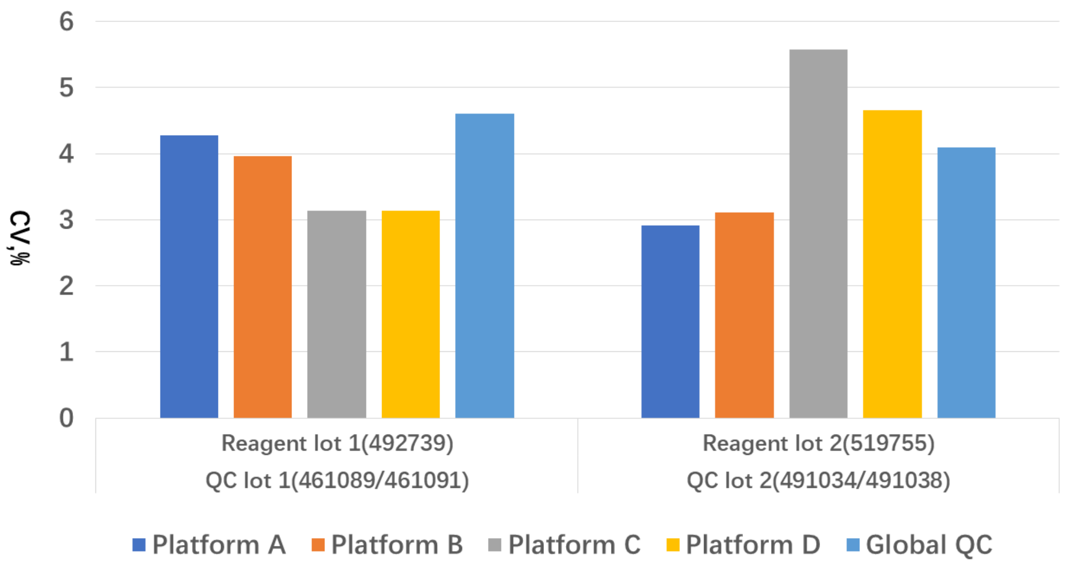

Supplementary Figure 3 CV for AHCV positive QC material and reagent lot partition for each instrument over a 6-month interval

The CV variation between two reagent lots of the same instrument for AHCV-positive QCs was 1.36-1.91-fold, 1.27-1.64-fold for the four instruments between the same reagent lot, and 1.91-fold for different reagent lots and different instruments. The CVs of instrument C and instrument D reagent lot 2 were higher than the CVs of the global QC data of the same lot.

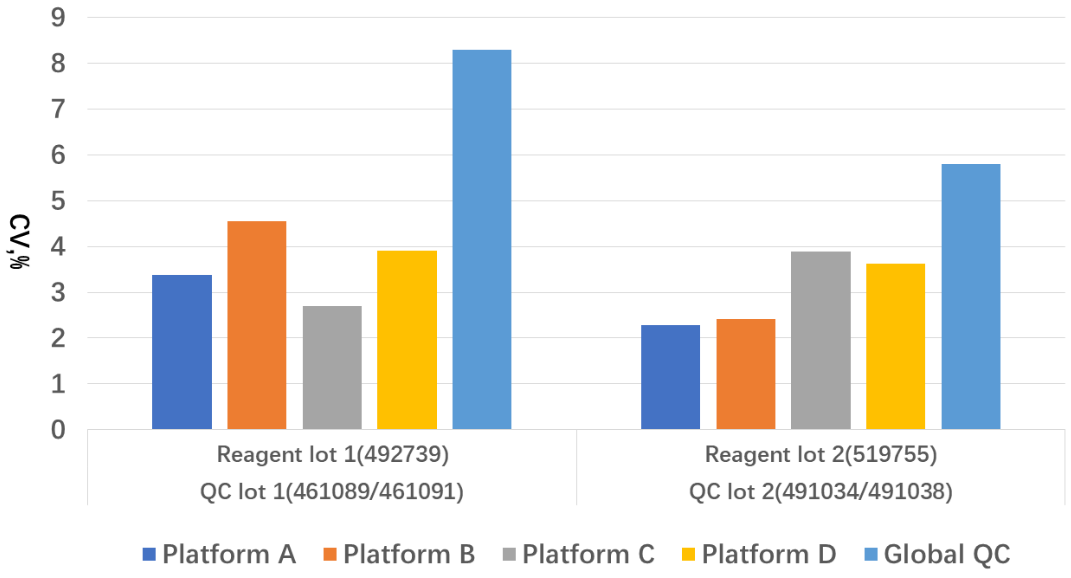

Supplementary Figure 4 CV for AHCV negative QC material and reagent lot partition for each instrument over a 6-month interval

The CV difference between two reagent lots for the same instrument for the AHCV

negative QC was 1.69-1.70-fold, 1.07-1.89-fold for the four instruments between the same reagent lots, and 1.99-fold for different reagent lots and different instruments. The CVs of all instruments were lower than the CVs of the global QC data of the same lot.

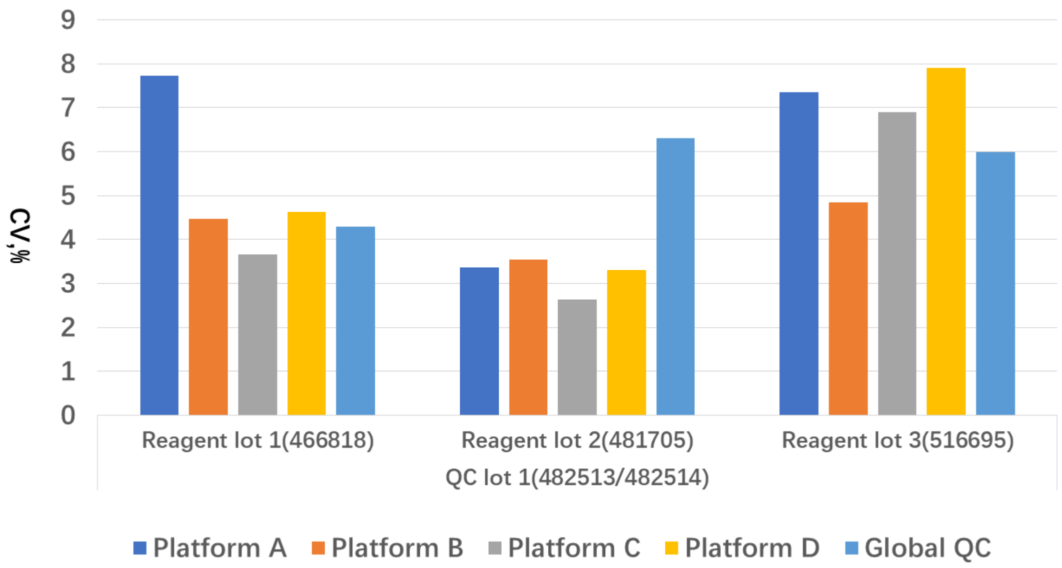

Supplementary Figure 5 CV for A-TP positive QC material and reagent lot partition for each instrument over a 6-month interval

The CV differences were 1.35-2.10-fold between three reagent lots for the same instrument for A-TP positive QC, 1.37-2.63-fold between the same reagent lots for the four instruments, and 3.00-fold between different reagent lots and different instruments. The CVs of reagent lot 1 and reagent lot 3 for instrument A, and reagent lot 3 for instruments C and D were higher than the CVs of the global QC data for the same lot.

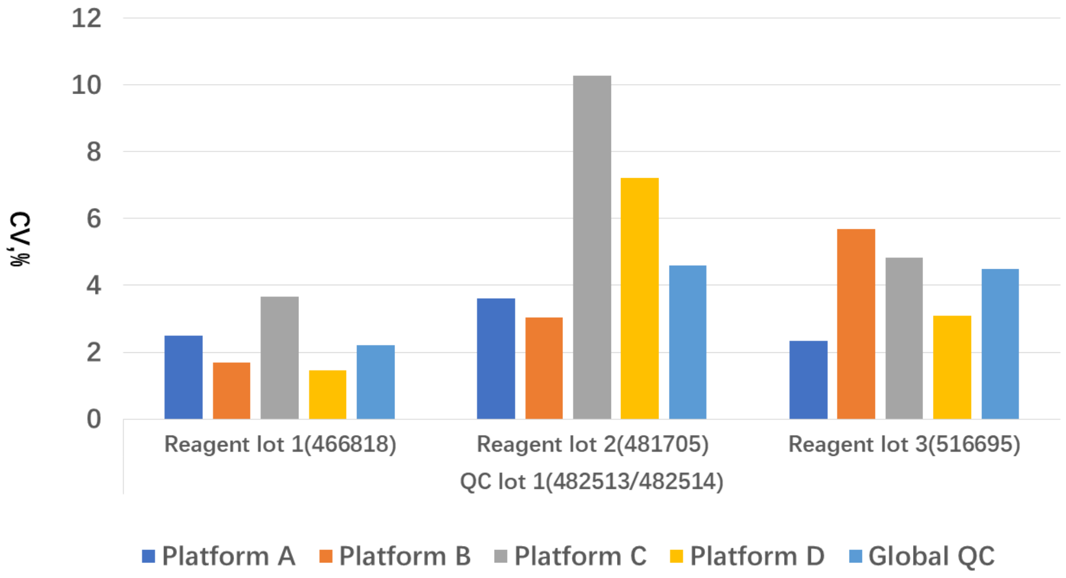

Supplementary Figure 6 CV for A-TP negative QC material and reagent lot partition for each instrument over a 6-month interval

The CV variation was 2.42-3.37-fold between three reagent lots for the same instrument for A-TP negative QC, 1.53-4.95-fold between the same reagent lots for the four instruments, and 7.05-fold between different reagent lots and different instruments. The CVs for reagent lot 1 of instrument A, reagent lot 3 of instrument B, all reagent lots of instrument C, and reagent lot 2 of instrument D were higher than the CVs for the same lot of global QC data.

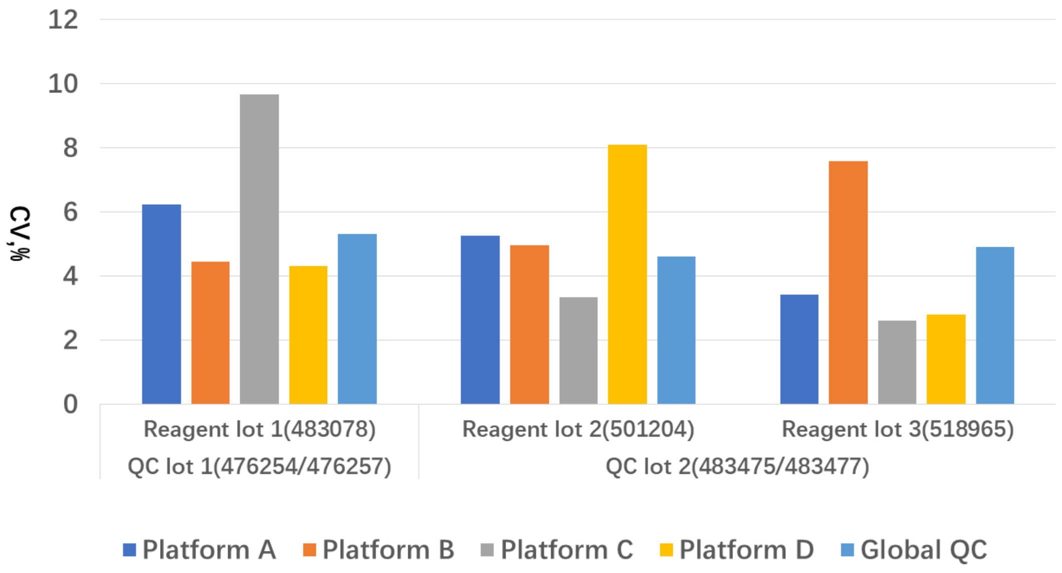

Supplementary Figure 7 CV for HIVAg positive QC material and reagent lot partition for each instrument over a 6-month interval

The CV variation was 2.25-2.92-fold between three reagent lots for the same instrument for HIVAg -positive QC, 1.71-3.72-fold between the same reagent lot for the four instruments, and 3.72-fold between different reagent lots and different instruments. The CVs of reagent lots 1 and 2 of instrument A, reagent lots 2 and 3 of instrument B, reagent lot 1 of instrument C, and reagent lot 2 of instrument D were higher than the CVs of the same lot of global QC data.

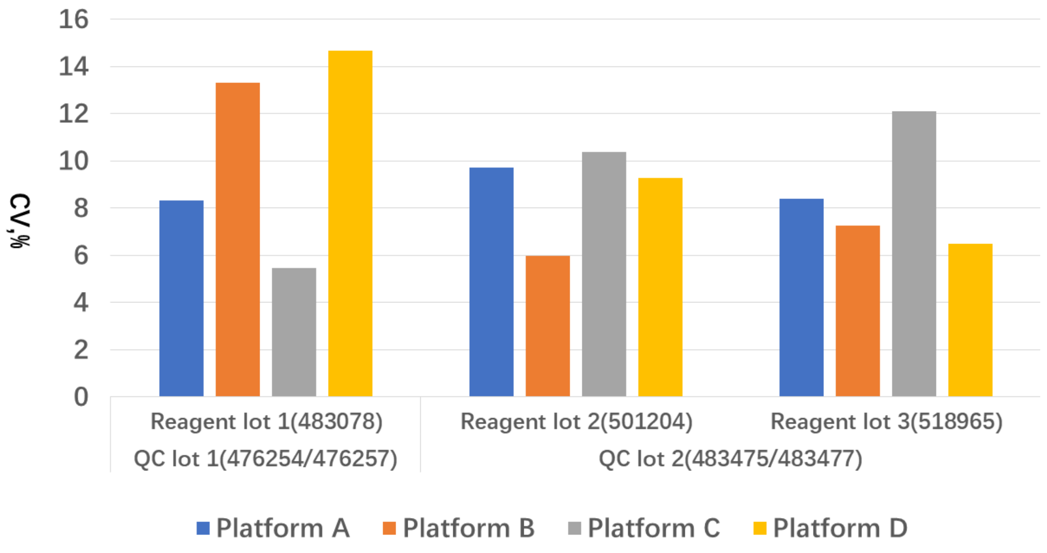

Supplementary Figure 8 CV for HIVAg negative QC material and reagent lot partition for each instrument over a 6-month interval

The CV variation was 1.73-2.68 times between three reagent lots of the same instrument for HIVAg negative QC, 1.17-2.25 times between batches of the same reagent for four instruments, and 2.68 times for different reagent lots and different instruments.

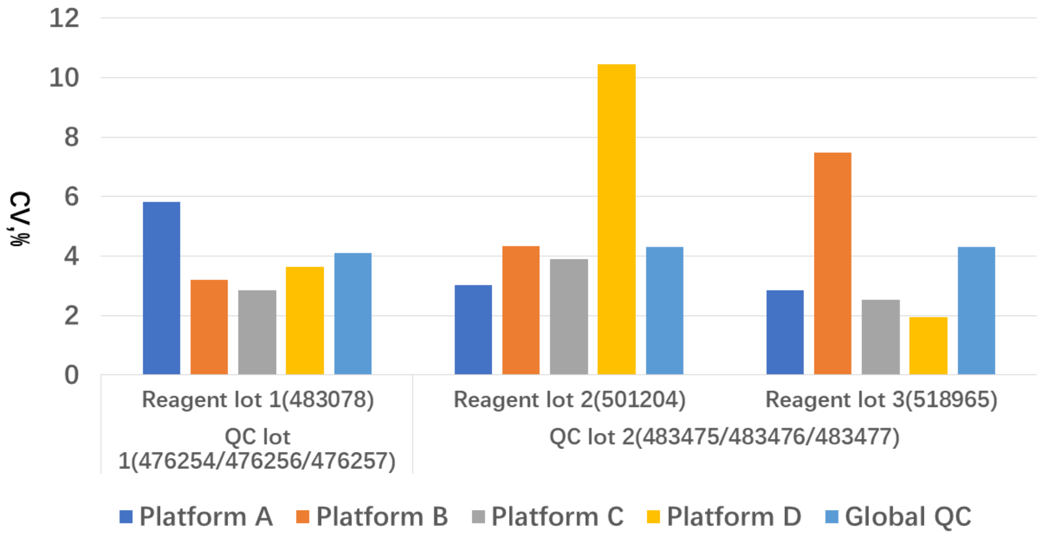

Supplementary Figure 9 CV for AHIV positive QC material and reagent lot partition for each instrument over a 6-month interval

The CV differences were 2.05-3.83-fold between three reagent lots of the same instrument for AHIV positive QCs, 1.53-5.36-fold between the same reagent lot for four instruments, and 5.36-fold between different reagent lots and different instruments. The CVs of reagent lot 1 of instrument A, reagent lots 2 and 3 of instrument B, and reagent lot 2 of instrument D were higher than the CVs of the same lot of global QC data.

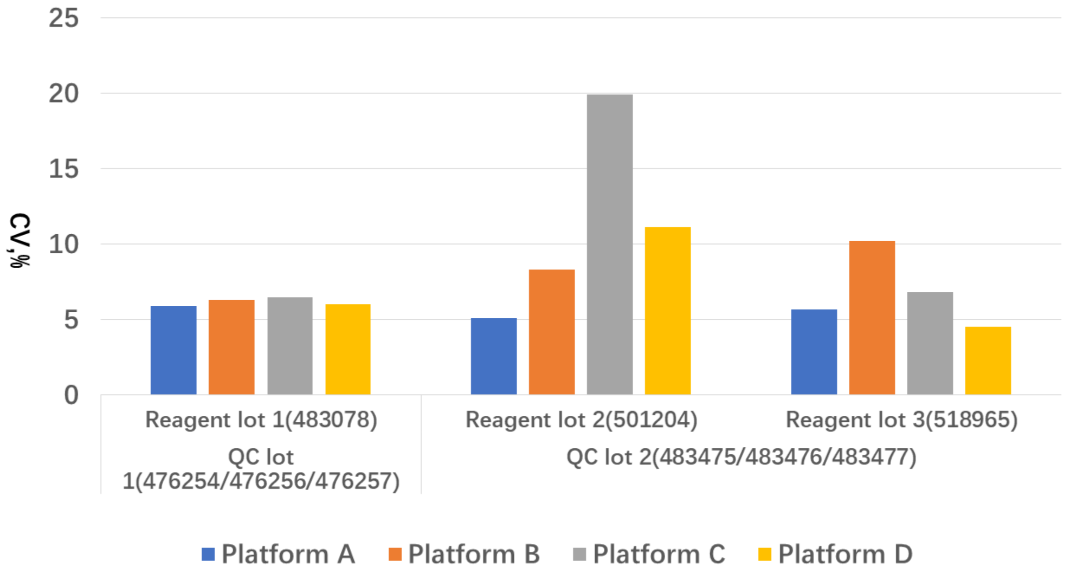

Supplementary Figure 10 CV for AHIV negative QC material and reagent lot partition for each instrument over a 6-month interval

The CV differences were 1.09-3.91-fold between three reagent lots of the same instrument for the AHIV negative QC, 1.16-3.08-fold between batches of the same reagent for the four instruments, and 4.39-fold for different reagent lots and different instruments.
