# Supplementary material for: A modified quality control protocol for infectious disease serology based on the Westgard rules
Source: Sci Rep. 2024 Jul 19;14:16683. doi: 10.1038/s41598-024-67472-1 (PMC11271505; doi:10.1038/s41598-024-67472-1)
Supplement: Supplementary file 2 — Supplementary Tables. [file 41598_2024_67472_MOESM2_ESM.pdf]

Supplementary Table 1 QC protocols included in the study

	protocol 1	protocol 2	Asymmetric Protocol
How mean value and SD determine	First 20 QC results to set $\bar{x}$ and SD	First 15 QC results to get $\bar{x}$ and Δ max	First 15 QC results to set $\bar{x}$ and SD
rejection limits	At least one of 1-3s、2-2s、R-4s occurred	Exceeding $\bar{x} \pm \Delta$ max	Negative QC results: Exceeding $\bar{x} \pm \Delta$max<br>Positive QC results: At least one of 1-3s 、 2-2s 、 R-4 occurred
Timing to re-establishment control limits	QC material lot change	QC material lot change	QC material or reagent lot change

Note:

① $\bar{x} = \frac{\sum_{i=1}^{n} x_i}{n}$;

② $SD = \sqrt{\frac{\sum_{i=1}^{n}(x_i - \bar{x})^2}{n}}$;

③ $CV = \frac{SD}{mean}$;

④ $\Delta_{max} = \sqrt{k^2 * s_{ep}^2}$ （*K=3*）;

Supplementary Table 2 QC materials and standard materials

	QC materials		Standard substances	
	manufacturer	level	manufacturer	level
HBsAg	ROCHE	Undefined	Conchstein Biotechnology Co., Ltd	0.2IU/ml
A-TP		Undefined		21mIU/ml
AHCV		Undefined		0.05NCU/ml
HIVAg		Undefined		2.5U/ml
AHIV		Undefined		4NCU/ml

Supplementary Table 3 Summary of level and lots of QC and standard materials

	Amount of QC materials lots	Amount of QC materials results			Amount of standard materials lots	Amount of standard materials results		
		negative	positive	total		negative	positive	total
HBsAg	3	1027	1023	2505	1	696	690	1386
AHCV	2	1101	1095	2196	1	696	690	1386
TP	1	921	911	1832	1	595	595	1190
HIVAg	2	880	879	2646	1	647	647	1294
AHIV	2	880	885	2646	1	635	635	1270
Total	10	4809	4793	11825	5	3269	3257	6526

Supplementary Table 4 Simulated data set created for an assay with a COI of 1

Critical positive simulation data										Critical negative simulation data									
modelling parameter：1.00-1.20							times：200			modelling parameter：0.80-1.00							times：200		
1.055	1.170	1.191	1.063	1.198	1.159	1.116	1.014	1.120	1.099	0.989	0.845	0.837	0.999	0.994	0.992	0.846	0.940	0.981	0.933
1.151	1.060	1.084	1.095	1.069	1.050	1.016	1.194	1.051	1.080	0.958	0.967	0.875	0.880	0.909	0.896	0.982	0.903	0.932	0.803
1.164	1.122	1.035	1.070	1.169	1.099	1.165	1.190	1.085	1.191	0.828	0.948	0.884	0.838	0.985	0.890	0.922	0.912	0.970	0.906
1.018	1.172	1.132	1.164	1.119	1.112	1.014	1.121	1.087	1.111	0.946	0.908	0.974	0.884	0.920	0.993	0.919	0.979	0.984	0.936
1.091	1.116	1.164	1.038	1.056	1.118	1.168	1.108	1.118	1.037	0.930	0.805	0.981	0.920	0.846	0.910	0.990	0.829	0.872	0.900
1.127	1.077	1.079	1.197	1.182	1.090	1.121	1.102	1.154	1.170	0.882	0.814	0.958	0.986	0.910	0.930	0.935	0.837	0.968	0.918
1.082	1.071	1.060	1.122	1.192	1.133	1.152	1.065	1.028	1.028	0.874	0.890	0.856	0.889	0.984	0.907	0.897	0.908	0.833	0.823
1.044	1.182	1.146	1.010	1.104	1.040	1.089	1.002	1.058	1.199	0.997	0.852	0.902	0.905	0.873	0.927	0.917	0.810	0.961	0.869
1.138	1.101	1.078	1.052	1.123	1.160	1.181	1.019	1.116	1.023	0.899	0.804	0.959	0.882	0.839	0.909	0.950	0.857	0.970	0.844
1.029	1.101	1.198	1.040	1.154	1.135	1.127	1.064	1.098	1.136	0.966	0.912	0.856	0.972	0.855	0.841	0.835	0.870	0.993	0.895
1.001	1.002	1.140	1.135	1.033	1.147	1.139	1.016	1.186	1.153	0.979	0.949	0.917	0.866	0.943	0.914	0.928	0.838	0.994	0.955
1.154	1.189	1.160	1.163	1.083	1.180	1.100	1.077	1.012	1.009	0.804	0.895	0.913	0.905	0.942	0.886	0.815	0.997	0.824	0.808
1.169	1.085	1.004	1.170	1.177	1.117	1.086	1.102	1.187	1.080	0.923	0.982	0.972	0.872	0.834	0.894	0.932	0.972	0.810	0.834
1.144	1.139	1.123	1.063	1.145	1.162	1.023	1.028	1.036	1.152	0.922	0.949	0.803	0.885	0.952	0.922	0.915	0.869	0.999	0.844
1.133	1.111	1.021	1.188	1.040	1.087	1.110	1.058	1.080	1.176	0.940	0.847	0.941	0.912	0.945	0.908	0.869	0.865	0.962	0.925
1.199	1.026	1.034	1.085	1.022	1.153	1.122	1.018	1.131	1.007	0.881	0.800	0.966	0.911	0.836	0.958	0.892	0.861	0.855	0.931
1.088	1.187	1.172	1.036	1.180	1.050	1.082	1.070	1.053	1.045	0.850	0.964	0.899	0.938	0.925	0.839	0.886	0.879	0.998	0.915
1.101	1.076	1.046	1.167	1.181	1.111	1.073	1.174	1.097	1.121	0.927	0.810	0.995	0.896	0.951	0.821	0.850	0.843	0.903	0.804
1.087	1.113	1.002	1.177	1.084	1.062	1.004	1.006	1.130	1.072	0.963	0.950	0.962	0.868	0.956	0.937	0.998	0.903	0.890	0.994
1.167	1.142	1.153	1.186	1.160	1.152	1.053	1.021	1.036	1.116	0.911	0.922	0.982	0.824	0.909	0.906	0.890	0.949	0.812	0.818

Supplementary Table 5 Simulated data set created for an assay with a COI of 0.9

Critical positive simulation data										Critical negative simulation data									
modelling parameter：0.90-1.08							times：200			modelling parameter：0.80-1.00							times：200		
0.918	1.026	0.965	1.060	0.978	0.959	1.048	0.960	1.016	1.029	0.763	0.742	0.880	0.853	0.868	0.758	0.796	0.880	0.767	0.720
0.982	1.033	0.993	1.013	0.978	0.960	1.044	1.011	0.994	1.052	0.847	0.898	0.831	0.807	0.726	0.897	0.788	0.867	0.747	0.781
0.930	0.940	1.054	0.938	0.953	0.924	0.949	1.017	0.960	0.939	0.824	0.754	0.794	0.833	0.773	0.895	0.760	0.732	0.730	0.859
1.064	0.970	1.056	0.984	0.957	0.932	0.911	1.037	1.033	0.937	0.727	0.736	0.790	0.743	0.724	0.867	0.757	0.748	0.756	0.852
1.052	0.957	1.060	1.079	0.958	1.006	0.954	1.032	0.901	0.969	0.767	0.838	0.894	0.733	0.742	0.738	0.762	0.739	0.885	0.860
1.005	1.066	1.043	0.905	1.049	1.001	1.016	1.014	0.947	0.989	0.861	0.789	0.814	0.843	0.736	0.825	0.739	0.888	0.814	0.767
0.929	0.981	0.938	0.907	0.902	1.067	0.959	1.040	0.977	0.977	0.749	0.866	0.740	0.857	0.837	0.767	0.804	0.878	0.796	0.770
0.966	1.027	1.059	0.953	0.929	1.051	1.060	0.973	0.969	1.014	0.743	0.813	0.840	0.816	0.783	0.724	0.795	0.879	0.753	0.779
0.991	0.957	1.033	0.966	1.033	0.911	0.997	0.919	1.061	0.921	0.830	0.797	0.754	0.793	0.761	0.772	0.738	0.815	0.741	0.858
1.012	0.930	0.937	0.909	1.070	0.907	0.990	0.968	0.983	1.016	0.807	0.752	0.739	0.739	0.857	0.812	0.727	0.819	0.785	0.805
1.034	1.018	0.937	1.004	1.071	0.918	1.037	1.001	0.937	1.064	0.890	0.762	0.773	0.893	0.884	0.774	0.880	0.758	0.899	0.812
0.938	0.949	0.905	1.066	0.956	1.009	0.989	0.928	1.049	1.023	0.849	0.772	0.758	0.778	0.892	0.818	0.900	0.852	0.729	0.852
1.075	0.913	1.001	0.995	1.059	0.993	0.950	1.077	0.953	1.026	0.777	0.813	0.830	0.810	0.843	0.812	0.771	0.752	0.779	0.732
1.023	1.002	0.904	1.080	0.968	1.003	1.041	0.911	1.045	1.067	0.741	0.898	0.830	0.819	0.781	0.793	0.778	0.855	0.845	0.821
1.026	0.978	1.035	1.020	0.943	0.974	1.053	0.981	0.962	0.927	0.784	0.837	0.820	0.734	0.897	0.744	0.859	0.897	0.872	0.825
0.954	0.934	1.046	0.913	0.931	0.946	1.068	0.906	0.998	0.918	0.883	0.765	0.857	0.851	0.766	0.884	0.793	0.720	0.811	0.818
1.029	0.921	1.009	0.931	1.003	1.079	0.930	1.024	1.057	1.051	0.798	0.854	0.894	0.869	0.767	0.844	0.897	0.810	0.738	0.817
1.041	0.986	1.015	1.033	1.078	0.971	1.010	0.909	0.994	0.903	0.885	0.867	0.817	0.832	0.894	0.814	0.731	0.842	0.788	0.812
0.935	0.953	1.045	0.980	1.042	0.993	1.021	1.051	1.063	0.957	0.892	0.739	0.875	0.859	0.834	0.796	0.754	0.836	0.813	0.788
1.073	1.010	0.999	1.006	0.912	0.928	0.957	1.042	0.991	0.969	0.853	0.829	0.816	0.857	0.899	0.769	0.873	0.746	0.763	0.725

Supplementary Table 6 nSD ranges of four instruments for each Analytes after the reagent lot changing

	nSD range			
	1st	2nd	3rd	4th
HBsAg-negative	-1.88～-4.85	0.57～3.99	-0.11～1.47	-1.28～2.36
HBsAg-positive	-0.22～3.08	1.40～2.98	-0.62～-1.78	-0.39～1.54
AHCV-negative	-0.07～3.81	-2.95～-4.17	----	----
AHCV-positive	-2.98～-4.58	-1.18～-2.58	----	----
TP-negative	-2.95～-5.81	----	----	----
TP-positive	0.86～3.27	----	----	----
HIVAg-negative	-0.02～-1.91	-2.28～0.71	----	----
HIVAg-positive	1.79～7.10	-0.04～1.30	----	----
AHIV-negative	3.87～6.94	-1.37～0.72	----	----
AHIV-positive	1.48～7.71	-1.72～1.89	----	----

----：Not involved

Supplementary Table 7 Pfr of different instruments for each analysis

	Pfr%(number)				P value
	Cobas 801A	Cobas 801B	Cobas 801C	Cobas 801D	
HBsAg	54.89(303)a	36.86(181)b	21.52(105)c	12.52(65)d	<0.05
AHCV	60.21(344)a	63.12(344)a	42.20(220)b	48.40(273)b	<0.05
TP	51.23(195)a	45.80(169)a	49.51(252)a	54.53(313)a	0.064
HIVAg	28.85(148)a	37.11(256)b	47.21(339)a	51.06(384)a	<0.05
AHIV	37.61(176)a	27.10(190)b	37.41(269)a	38.39(291)a	<0.05
P value	<0.05	<0.05	<0.05	<0.05	

Note: Analytes with different subsequent letters of Pfr were considered statistically significant differences at the 0.05 level

Supplementary Table 8 CV for the different quantities of QC data

Number of QC data	CV(%)		
	HBsAg-Positive	AHCV- Positive	TP- Positive
15	3.32	2.44	1.79
20	3.17	2.65	1.61
30	3.13	2.56	1.55
40	3.32	2.49	1.43
60	3.83	2.14	1.44
80	3.73	2.93	1.33
100	3.85	3.12	2.52

Supplementary Table 9 Pfr of rejection limits in traditional QC rules and new QC rule models

	Instrument	Number of results	Pfr(%) Protocol 1	Protocol 2	Asymmetric Protocol	P value
HBsAg-positive	A	168	29.17a	22.02b	4.17c	<0.05
	B	185	25.41a	12.97b	0c	<0.05
	C	168	26.19a	23.81a	1.80b	<0.05
	D	175	27.43a	22.86a	5.49b	<0.05
HBsAg-negative	A	168	8.33a	1.79b	0.60b	<0.05
	B	179	27.37a	14.53b	0c	<0.05
	C	168	24.40a	9.52b	0c	<0.05
	D	175	0.57a	2.86a	0a	<0.05
A-HCV-positive	A	168	47.02a	24.40b	40.85a	<0.05
	B	185	54.86a	31.94b	24.82b	<0.05
	C	168	69.68a	44.52b	55.78a,b	<0.05
	D	175	26.54a	6.17b	16.46a	<0.05
A-HCV-negative	A	168	47.62a	23.81b	28.05b	<0.05
	B	179	25.69a	18.06a,b	12.06b	<0.05
	C	168	32.26a	28.39a	22.76a	0.273
	D	175	38.89a	26.54a	31.68a	0.055
A-TP-positive	A	142	52.11a	36.62a,b	31.43b	<0.05
	B	149	24.83a	6.71b	26.71a	<0.05
	C	145	22.07a	6.90b	13.53a,b	<0.05
	D	159	14.79a	6.34a	14.88a	0.077
A-TP-negative	A	142	69.01a	58.45b	15.49c	<0.01
	B	149	72.6a	69.18a	37.67b	<0.01
	C	145	60.69a	46.21a	0b	<0.01
	D	159	45.07a	51.41b	0c	<0.01
HIV Ag-positive	A	166	20.48a	9.64a	22.29a	0.051
	B	166	14.46a	20.48a,b	30.72b	<0.01
	C	157	4.46a	20.38b	29.38b	<0.05
	D	158	22.01a	36.48a,b	49.69b	<0.01
HIV Ag-negative	A	166	52.41a	36.14b	32.53b	<0.01
	B	166	28.92a	15.06b	12.82b	<0.01
	C	157	14.65a	8.92b	19.75a	0.024
	D	158	26.42a	25.79a	21.88a	0.084
A-HIV-positive	A	149	13.42a	9.40b	8.72b	0.022
	B	155	10.97a	18.71a	0b	<0.01
	C	156	24.36a	14.74a	26.92a	0.054
	D	175	40.51a	42.41a	60.76b	<0.01
A-HIV-negative	A	149	9.40a	2.01a	5.37a	0.28
	B	155	9.68a	9.03a	9.67a	0.064
	C	156	8.33a	3.85b	4.49a	<0.01
	D	175	1.27a	6.96b	1.27a	<0.01

Note: Analytes with different subsequent letters (a,b, and c) of Pfr were considered statistically significant differences at the 0.05 level

Supplementary Table 10 Ped of rejection limits in traditional QC rules and new QC rule models

	Instrument	Number of results	protocol 1	protocol 2	Asymmetric Protocol
HBsAg-positive	A	200	100	100	100
	B	200	100	100	100
	C	200	100	100	100
	D	200	100	100	100
HBsAg-negative	A	200	100	100	100
	B	200	100	100	100
	C	200	100	100	100
	D	200	100	100	100
A-HCV-positive	A	200	100	100	100
	B	200	100	100	100
	C	200	100	100	100
	D	200	100	100	100
A-HCV-negative	A	200	100	100	100
	B	200	100	100	100
	C	200	100	100	100
	D	200	100	100	100
A-TP-positive	A	200	100	100	100
	B	200	100	100	100
	C	200	100	100	100
	D	200	100	100	100
A-TP-negative	A	200	100	100	100
	B	200	100	100	100
	C	200	100	100	100
	D	200	100	100	100
HIV Ag-positive	A	200	100	100	100
	B	200	100	100	100
	C	200	100	100	100
	D	200	100	100	100
HIV Ag-negative	A	200	100	100	100
	B	200	100	100	100
	C	200	100	100	100
	D	200	100	100	100
A-HIV-positive	A	200	100	100	100
	B	200	100	100	100
	C	200	100	100	100
	D	200	100	100	100
A-HIV-negative	A	200	100	100	100
	B	200	100	100	100
	C	200	100	100	100
	D	200	100	100	100
